# Pseudo‐2:1 bundle branch block. “Fusion causes confusion”

**DOI:** 10.1002/joa3.12940

**Published:** 2023-10-31

**Authors:** S. Serge Barold, Harry G. Mond

**Affiliations:** ^1^ University of Rochester School of Medicine and Dentistry Rochester New York USA; ^2^ The Royal Melbourne Hospital and University of Melbourne, Parkville and Cardioscan PTY LTD Camberwell Victoria Australia

**Keywords:** bundle branch block, end‐diastolic ventricular extrasystole, parasystole, ventricular fusion

## Abstract

The fusion of narrow‐QRS sinus‐generated beats with end‐diastolic ventricular extrasystoles occurring in bigeminy can produce an electrocardiographic pattern difficult to differentiate from parasystole. Such an ECG should not be interpreted as 2:1 RBBB because of the variability of the PR intervals.

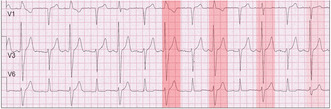

The fusion of narrow‐QRS sinus‐generated beats with end‐diastolic ventricular extrasystoles occurring in bigeminy may produce electrocardiographic (ECG) patterns resembling 2:1 bundle branch block or parasystole.

Figure 1 shows sinus rhythm, first degree atrioventricular (AV) block (PR interval ~ 280 ms) with 2:1 right bundle branch block configuration (RBBB) with the QRS duration gradually becoming shorter. The RBBB beats alternate with relatively narrow QRS complexes. The ECG diagnosis of 2:1 RBBB requires that all PR intervals of fully conducted beats be equal. However, this is not the case because the PR intervals of the beats with an RBBB configuration, range between 140 ms and 200 ms. The RBBB complexes represent “end diastolic” ventricular ectopic beats. The varying PR intervals result from minor changes in the sinus rate and slight changes in the timing of the extrasystolic beats resulting in various degrees of fusion with normally conducted sinus beats. This results in varying QRS configurations and widths (red highlight gradually becoming lighter). The duration of the PR intervals of the fusion beats gradually lengthen so that the contribution of the sinus beats increases. This is clearly seen in the last fusion beat with a PR interval close to 200 ms and a QRS configuration resembling that of a normally conducted beat. This ECG should not be interpreted as a 2:1 RBBB block with the interspersed normally conducted QRS complexes suggesting intermittent improved conduction in the right bundle branch. Preexcitation can also be ruled by the marked variability of the PR intervals.

**FIGURE 1:**
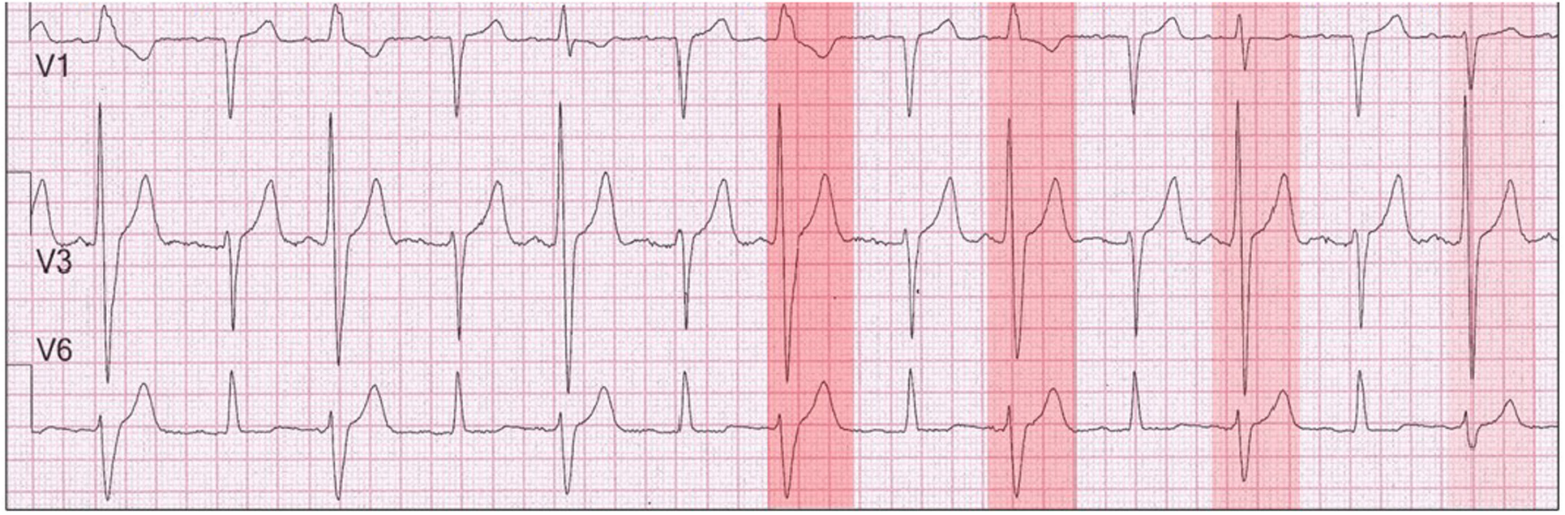
Pseudo‐2:1 RBBB. V1 shows sinus rhythm with alternating RBBB beats. These are end‐diastolic ventricular extrasystoles that fall within the PR interval of the next sinus complex. When the extrasystoles occur later, the PR interval lengthens and there is less contribution of the extrasystole to the fused QRS complex. The first highlight is the darkest and shows no fusion. The highlights lighten as the percentage of sinus involvement increases.

The presence of slight coupling variations of the extrasystoles to the preceding QRS complex and the presence of fusion suggest the diagnosis of ventricular parasystole. However, this possibility is unlikely because the extrasystoles are not evenly spaced showing slight variations of the inter‐ectopic intervals.^1^ This case illustrates the difficulty of differentiating fusion induced by end‐diastolic extrasystoles from that associated with parasystole. A more striking form of pseudo‐2:1 BBB may also occur when the underlying rhythm is sinus with BBB.^2^ In this situation, when end‐diastolic ventricular extrasystoles with fixed coupling in bigeminy fusing with a sinus‐conducted beat, the resultant fusion beat may be narrow and simulate a normally conducted beat.

Figure 2 shows end‐diastolic ventricular extrasystoles (red highlight gradually becoming lighter) fuse with sinus‐conducted beats as their coupling intervals lengthen. The first ventricular ectopic beat (VE) demonstrates no fusion because its coupling interval is shorter than that of the subsequent intervals. Longer coupling intervals of the ventricular extrasystoles (V fusion) promote fusion with the sinus‐conducted beat. The inter‐ectopic intervals are constant suggesting the presence of parasystole rather than simple fusion from late ventricular extrasystoles. A firm diagnosis of parasystole would require longer ECG recordings.

**FIGURE 2:**
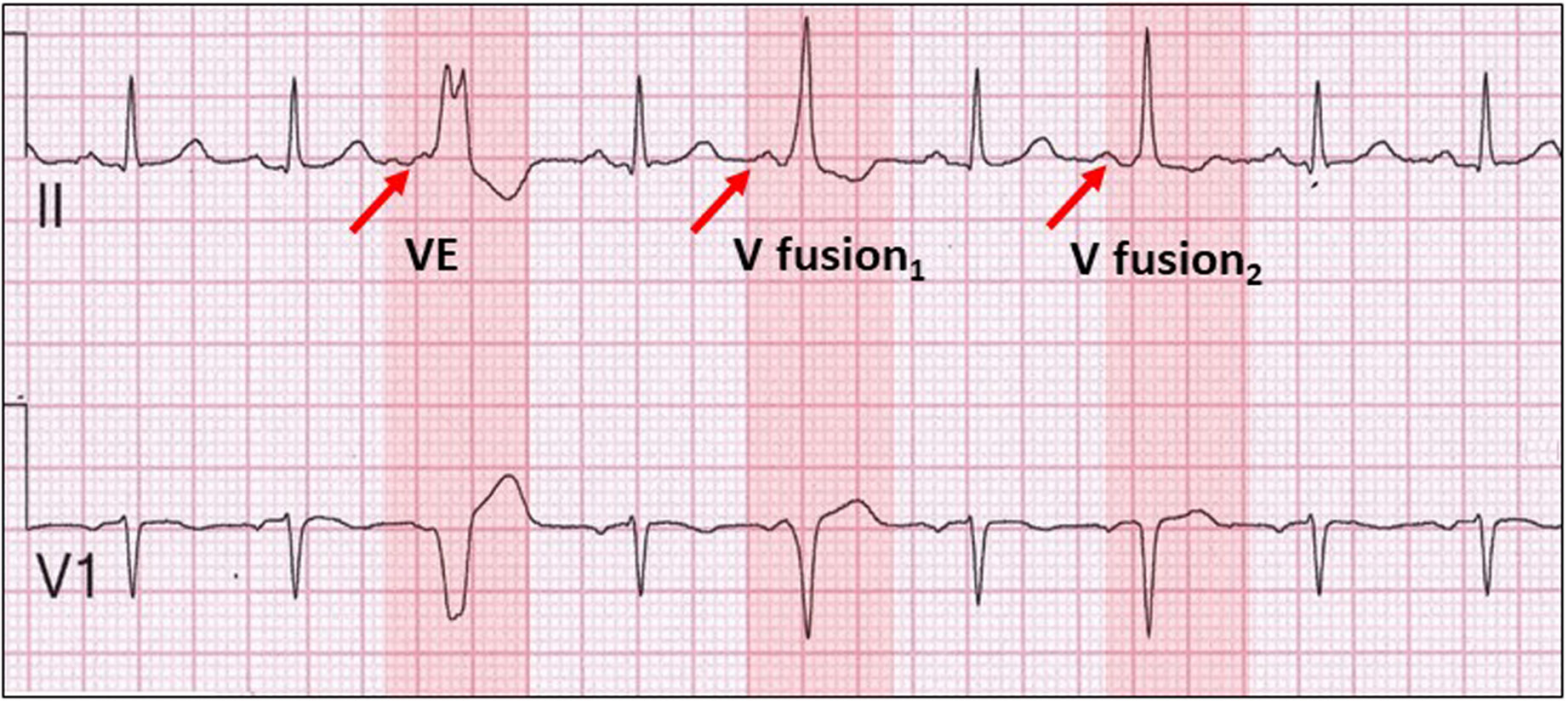
Pseudo‐2:1 left bundle branch block. See text for details.

## CONFLICT OF INTEREST STATEMENT

Authors declare no conflict of interests for this article.
